# Oxidative conversion of lignin isolated from wheat straw into aromatic compound catalyzed by NaOH/NaAlO_2_

**DOI:** 10.1002/fsn3.1633

**Published:** 2020-05-22

**Authors:** Ke‐Hui Luo, Si‐Jiu Zhao, Guo‐Zhi Fan, Qun‐Peng Cheng, Bo Chai, Guang‐Sen Song

**Affiliations:** ^1^ School of Chemical and Environmental Engineering Wuhan Polytechnic University Wuhan China

**Keywords:** aromatic compound, composite catalyst, lignin, oxidative conversion, Wheat straw

## Abstract

Lignin was isolated from wheat straw via organosolv process and further transferred to monophenolic compounds via oxidative conversion. Wheat straw lignin (WSL) with purity at 91.4 wt% was acquired in the presence of heterogeneous and recyclable catalyst of Amberlyst‐45. WSL was characterized by infrared spectrometer (IR), nuclear magnetic resonance spectroscopy (NMR) including ^1^H NMR and ^13^C NMR spectra. The results showed that WSL possesses typical syringyl (S), guaiacyl (G), and *p*‐hydroxyphenyl (H) units, and it is mainly composed of S and G units. The product distribution was dependent on the composition of WSL. Derivatives from S and G units were found to be the main products. The oxidative conversion of WSL was performed by varying oxidant and catalyst. Both the formation of monophenolic compounds and aromatic aldehydes were enhanced by combining oxidants and catalysts. The composite catalyst composed of NaOH/NaAlO_2_ was effective for the oxidation of WSL in the presence of nitrobenzene and atmospheric pressure air. The total yield of monophenolic compounds reached up 18.1%, and yields at 6.3 and 5.7% for syringaldehyde and vanillin were achieved, respectively.

## Graphical abstract

The oxidative conversion of lignin isolated from wheat straw into aromatic compounds was investigated. Wheat straw lignin with high purity at 91.4 wt% was first isolated via organosolv process in the presence of green and recyclable catalyst of Amberlyst‐45. The oxidative conversion of lignin was then performed using NaOH/NaAlO_2_ as composite catalyst, and the total yield of monophenolic compounds reached 18.1%.

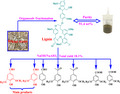

## 1 INTRODUCTION

Fossil fuels are being quickly consumed because of overexploitation resulted from a large increase in human population and industrial development. The worldwide energy crisis and environmental impact have caused extensive research and development programs on the conversion of lignocellulose to produce chemicals and biofuels (Besson, Gallezot, & Pinel, 2014;Cheng et al., 2020;Nanda, Reddy, Vo, Sahoo, & Kozinski, 2018;Peng, Yao, Zhao, & Lercher&, 2012;Tsegaye, Balomajumder, & Roy, 2020). Lignin is the most important component in the support tissues of lignocellulose (Martone et al., 2009). It is also the second most abundant natural plant material of biomass, accounting for nearly 30% of the organic carbon on Earth (Hage et al., 2009). Thus, lignin is expected to play an important role in the production of bio‐products in the near future (Gasser, Hommes, Schaffer, & Corvini, 2012).

Lignin is a hetero‐aromatic biopolymer composed of three primary monomers: sinapyl, coniferyl, and *p*‐coumaryl alcohols, joining by ether and C − C linkages. The monolignols produce *p*‐hydroxyphenyl (H), guaiacyl (G), and syringyl (S) units when they are incorporated into lignin polymer (Caballero, Font, & Marcilla, 1996). Lignin is the only renewable nonpetrochemical resource for aromatic compounds in nature (Choi et al., 2019). So it is considered a potential feedstock for renewable aromatic compounds, and the interest in the conversion of lignin to monophenolic compounds is growing. Generally, the depolymerization of lignin offers small segments such as monomeric phenols and oligomers (Fan et al., 2019;Li, Zhao, Wang, Huber, & Zhang, 2015;Rahimi, Ulbrich, Coon, & Stahl, 2014). However, lignin is still underused, and around 5% lignin is employed in low‐value commercial applications so far (Hu, Pan, Zhou, & Zhang, 2011). The conversion of lignin is still challenging due to its three‐dimensional amorphous polymer structure and complex composition.

The oxidative conversion of lignin is a noticeable way (Fargues, Mathias, & Rodrigues, 1996), in which the breakage of chemical linkages is achieved by oxidative depolymerization, generating valuable aromatic chemicals and/or providing a source of low‐molecular‐mass feedstocks for downstream processing (Ragauskas et al., 2014). The oxidative cracking includes the cleavage of aryl ether bonds, C − C bonds, aromatic rings, or other linkages of lignin polymer. Nitrobenzene (Sun, Lawther, & Banks, 1995), metal oxides (Voitl, & Von Rohr, 2008), molecular oxygen (Aarabi, Mizani, & Honarvar, 2017;Voitl, Nagel, & Von Rohr, 2010), and hydrogen peroxide (Su, Yang, Liu, & Lin, 2014) are the most regular oxidants. Homogenous catalytic systems such as salen complex (Badamali, Luque, Clark, & Breeden, 2009;Biannic, Bozell, & Elder, 2014), metal complex (Hanson, Wu, & Silks, 2012), oxide (Das, Kolar, Sharma‐Shivappa, Classen, & Osborne, 2017), base (Azarpira, Ralph, & Lu, 2014), and acid catalyst (Voitl et al., 2010;Voitl, & Von Rohr, 2009;Werhan, Mir, Voitl, & Von Rohr, 2011) have been developed to convert lignin into valuable chemicals. The most valuable products from lignin oxidation are monophenolic compounds including syringaldehyde, syringic acid, vanillin, vanillic acid, *p*‐hydroxybenzaldehyde, and *p*‐hydroxybenzoic acid (Partenheimer, 2009). Alkaline oxidation of lignin is considered the only approach that yields large amounts of single monomeric aldehydes. Aldehyde compounds are the predominant products in the presence of base catalysts, in which vanillin is usually the most readily available (Besson et al., 2014). The yield of aldehyde depends on the source and the pretreatment of lignin extremely (Voitl, Nagel, & Von Rohr, 2010). However, strong alkali such as NaOH is often used in the alkaline oxidation, resulting in problems of corrosion and pollution. The oxidation of lignin in nonalkaline media such as formic acid aqueous solution (Rahimi et al., 2014), mixture of alcohol and water has also been developed (Voitl, & Von Rohr, 2008, 2009). The formation of methyl vanillate is considerable while less aromatic aldehydes are produced.

It is well‐known that there is an interaction among the three main constituents including cellulose, hemicellulose, and lignin due to complex structure of lignocellulose. The direct conversion of lignocellulose often supplies poor yield and complex composition of product, leading to difficulty in separation. Therefore, effective isolation of the main constituents is a key operation for obtaining convertible raw materials including lignin and cellulose. Straw is a common and easily available lignocellulose, making up about half of the yield of cereal crops such as rice, wheat, and corn. China is the largest agricultural country in the world, around 900 million tons of straw is output annually. It is of great significance to develop comprehensive utilization of straw resources as the shortage of fossil resources becomes more and more serious. Wheat straw is one of the most important crop stalks, and it possesses high lignin content (Ramezani & Sain, 2018). Thus, it is expected to play an important role for lignin application. Some detailed works related to the treatment of wheat straw have been reported (Tian, Zhao, & Chen, 2018). Thereinto organosolv fractionation is extensively used to separate the three main components because it aims to fractionate lignocellulosic biomass into its natural structure as much as possible, in which the hydrolysis of hemicellulose and the dissolution of lignin occurs. Inorganic strong acid of sulfuric acid is usually used to promote the hydrolysis of hemicellulose and thus promoting the separation of constituents.

The aim of this work is to investigate an effective catalytic system for the oxidation of lignin isolated from wheat straw via a green organosolv process. The isolation of lignin was performed in the presence of recyclable solid acid of Amberlyst‐45. The structure of wheat straw lignin (WSL) was characterized by infrared spectroscopy (IR) and nuclear magnetic resonance (NMR). The oxidative conversion of lignin into monophenolic compounds was further investigated by varying oxidant and catalyst. The products derived from lignin were quantitatively and qualitatively analyzed by high‐performance liquid chromatography (HPLC), gas chromatography–mass spectrometry (GC‐MS), and liquid chromatography–high‐resolution mass spectrometry (LC‐HRMS), respectively.

## 2 MATERIALS AND METHODS

### 2.1 Materials

Wheat straw was supplied by local farm around Wuhan city (Hubei, China). Dealkaline lignin was purchased from J & K chemical. Other reagents were commercially purchased from Sinopharm Chemical Reagent Co., Ltd and used without further purification. Wheat straw was extracted in the mixture of toluene/ethanol to remove soluble impurity according to the reported procedure (Fan et al., 2013), and the as‐obtained sample was denoted as extracted wheat straw (EWS). WSL was grinded and screened by 80‐mesh sieve before oxidative conversion. NaAlO_2_ was calcined under nitrogen atmosphere before reaction.

### 2.2 Organosolv fractionation of wheat straw

Typically, 10 g EWS, 100 ml 1, 4‐dioxane/H_2_O mixture (2:1, v/v), and 5 g Amberlyst‐45 were added into a 500‐ml stainless steel autoclave. The mixture was heated at 180°C for 2.5 hr under magnetic stirring. The mixture was then cooled to room temperature, followed by filtration and washing thoroughly using 25 ml 1, 4‐dioxane/H_2_O mixture thrice. The washes and filtrate were combined before the addition of 3 volumes of H_2_O. WSL was precipitated and collected after centrifugation and freeze‐drying. The contents of cellulose, hemicellulose, lignin as well as ash in the samples including original wheat straw and WSL were determined according to the reported procedures (Chen, Yu, Zhang, & Lu, 2011). Amberlyst‐45 was recovered by screening the filter residual through a 40‐mesh sieve.

### 2.3 Oxidative conversion of lignin

Typically, 1 g WSL in 50 ml 1 M NaOH aqueous solution was dispersed via ultrasonic vibration before adding to a 500 ml autoclave. Then, 4 ml nitrobenzene and 4.1 g NaAlO_2_ were added. The mixture was then heated at 170°C for 2.5 hr under magnetic stirring. The reactor was quickly cooled by placing in an iced water bath during 20 min. The reaction mixture was then acidified by 20 wt% hydrochloric acid until the pH value of 1. After filtration, the filtrate was extracted by chloroform to obtain low‐molecular‐weight compounds including monophenolic compounds and oligomers. The organic soluble fraction was then quantitatively analyzed by HPLC. Yield was calculated as following: product mass divided WSL mass.

### 2.4 Measurements

IR was carried out on an EQUINOX 55 spectrometer in the range from 4,000 to 400 cm^–1^. The solid samples were grounded with dried KBr powder and compressed into a disk prior to analysis. ^1^H NMR and ^13^C NMR spectra were acquired on a Unity Inova 600 at 50°C using DMSO‐d6 as solvent and tetramethylsilane as internal standard. The quantitative analysis of the reaction product was performed on a LC‐100 PLUS HPLC equipped with reversed‐phase Novapak‐C18‐100 silica column (4.6 × 150 mm, 4 μm) and UV detector. The mixture of acetonitrile: 1 wt% phosphoric acid solution (12:88, V/V) was employed as mobile phase at the flow rate of 1.2 ml/min with column temperature at 32°C. The product was also qualitatively analysis by LC‐HRMS and GC‐MS. LC‐HRMS was performed on a UFLC‐20A high‐performance liquid chromatography (Shimadzu) and AB SciexTripleTOF 5,600 mass spectrometer equipped with an Agilent XDB‐C18 (2.1 × 100 mm, 3.5 μm). GC‐MS was performed on a Varian 450 GC‐320 MS equipped with a VF‐5 MS capillary column (30 m × 0.32 mm × 0.25 μm).

## 3 RESULTS AND DISCUSSION

### 3.1 Characterization

#### 3.1.1 IR

**Figure 1:**
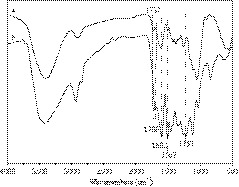
IR spectra of (a) EWS and (b) WSL

The IR spectra of EWS and WSL are presented in Figure 1. In the spectrum of EWS (curve 2a), the bands around 1737 and 1,157 cm^–1^ are assigned to the stretching vibration of aliphatic esters of hemicellulose and β–(1–4)–glycosidic bond of cellulose. These bands almost disappear in the spectrum of EWS (curve 2b), suggesting nearly no cellulose and hemicellulose in WSL after treating by organosolv process. The IR spectrum of WSL (curve 2b) presents typical characteristic bands at 1,602 and 1,507 cm^−1^ ascribed to aromatic ring vibrations (Bauer, Sorek, Mitchell, Ibáñez, & Wemmer, 2012). Both of them become stronger compared to those of EWS. The stretching vibration around 1706 cm^–1^ is ascribed to unconjugated ketones, carbonyls, ester groups, conjugated aldehydes, and carboxylic acids, which are possibly resulted from the cleavage of linkage in lignin polymer (Hage et al., 2009). These results confirm that lignin isolated from wheat straw with relatively high purity. It is believed to be beneficial to the oxidative conversion subsequently.

#### 3.1.2 NMR

**Figure 2:**
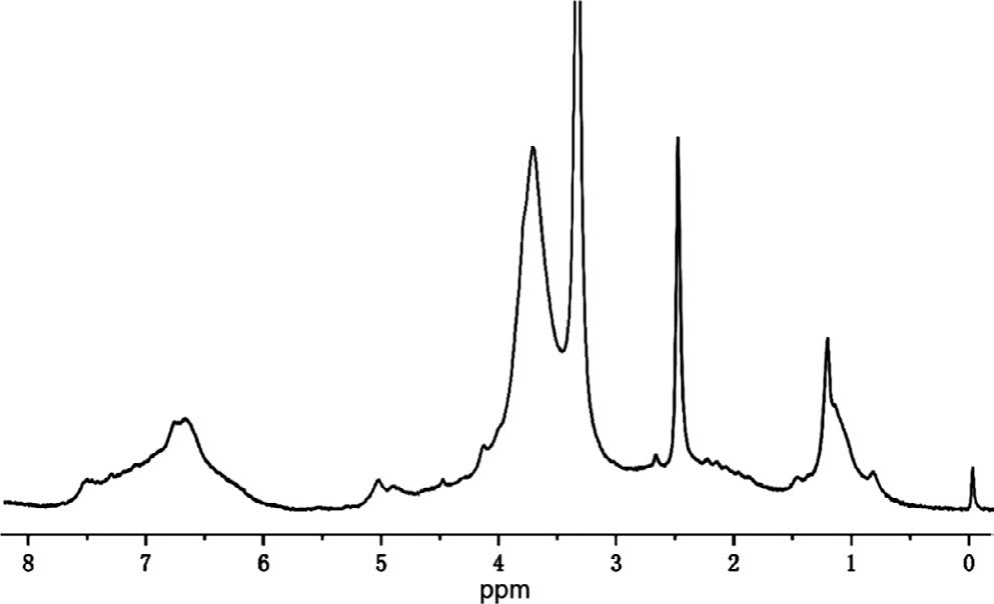
^1^H NMR spectrum of WSL

The ^1^H NMR spectrum of WSL is shown in Figure 2. The signals ascribed to H protons of *p*‐hydroxyphenyl units present around 7.46 ppm. The signals in the range from 7.12 to 6.40 ppm are assigned to aromatic protons in syringylpropane and guaiacylpropane structures (Ralph, Ralph, Landucci, & Landucci, 2004;Xu et al., 2008). The signal at 6.89 and 6.72 ppm are ascribed to aromatic protons in guaiacylpropane and syringylpropane structures, respectively (Jiang, 2008). The signals in the range from 3 to 5.8 ppm are ascribed to β–O–4 structure along with other linkages including β‐β and β‐5 (Ralph et al., 2004). The α‐H and β‐H in β‐O‐4 structures give signals in the range from 4.2 to 5.10 ppm. H protons of side chain give signals in the range from 4.50 to 4.0 ppm. Methoxyl protons (–OCH_3_) produce a strong signal at 3.67 ppm. These results reveal that WSL possesses typical G/S/H units with β–O–4 linkage. The content of H proton can be determined according to integral value. It can be seen from Figure 2 that WSL is mainly composed of S and G units.

**Figure 3:**
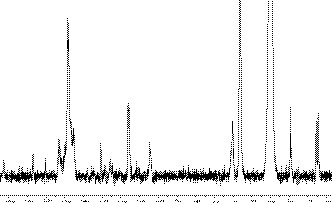
^13^C NMR spectrum of lignin isolated from wheat straw

Figure 3 presents ^13^C NMR spectrum of WSL. The characteristic signal of lignin can be seen in the range from 104 to 168 ppm. S units produce signals at 152 − 154 ppm (C_3_/C_5_, etherified), 148 ppm (C_3_/C_5_, nonetherified), 138 ppm (C_4_, etherified), 134.6 ppm (C_1_, etherified) and 104.3 ppm (C_2_/C_6_). G units produce signals at 150 ppm (C_3_, etherified), 148 ppm (C_4_, etherified), 145.6 ppm (C_3_/C_4_, nonetherified), 145 ppm (C_4_, nonetherified), 135.8 ppm (C_1_, etherified), 119.6 ppm (C_6_), and 111.7 ppm. Hydroxyphenyl units produce signal at 128.3 ppm (C_2_/C_6_) and 115.7 ppm (C_5_) (Hage et al., 2009;Xu et al., 2008;Xu, Yan, Dong, Liu, & Liu, 2012). The signals at 167 ppm (C_γ_), 160 ppm (C_4_), 130.7 ppm (C_2_/C_6_), 125.4 ppm (C_1_), and 116.2 ppm (C_3_/C_5_) belong to *p*‐coumaryl ester. The β − O−4 linkage is also detected by the observation of signals at 72.4 ppm (C_α_), 86.5 ppm (C_β_) and 60.3 ppm (C_γ_) (Xu, Geng, Liu, Ren, Sun, & Sun, 2008). The signal assigned to methoxy group of S and G units presents signal around 56.4 ppm. α and β methylene groups produce signal around 29 ppm. The signals in the range from 14 to 15 ppm belong to γ‐methyl in *n*‐propyl of side chain (Hage et al., 2009). In addition, no signals ascribed to saccharides appear in the range from 90 to 102 ppm (Deng et al., 2016), further indicating almost no cellulose and hemicellulose in WSL. It is in accordance with the IR spectra as shown in Figure 1. The ^13^C NMR spectrum further reveals that WSL is G/S/H type with high purity. The ^13^C NMR spectrum of dealkaline lignin (see supplementary material) reveals that it is quite similar to the spectrum of WSL except the absence of signals ascribed to *p*‐coumaryl ester and the observation of signal belonged to ferulic acid (Xu, Yan, Dong, Liu, & Liu, 2012). The signal of ferulic acid indicates that linkage cleavage of G units possibly occurs during the production process of dealkaline lignin. In addition, the signals of β − O−4 linkage in the spectrum of dealkaline lignin are rather weak, indicating that the β − O−4 linkage is little. These differences reveal that lignin structure depends on the source and the pretreatment conditions. Lignin separated from wheat straw via organosolv process keeps natural structure well.

### 3.2 Isolation of lignin from wheat straw

#### 3.2.1 Organosolv fractionation of wheat straw

Common solvents including methanol, ethanol, 1,4‐dioxane, acetonitrile, and acetone were usually used for the organosolv fractionation of wheat straw (Asadi & Zilouei, 2017), in which inorganic strong acids including H_2_SO_4_ or HCl are usually employed as catalyst. The yield and content of WSL is shown in Table 1. The results indicated that both the yield and content depended on organic solvent significantly (entries 1 − 5). Although ethanol was widely employed for organosolv process, yield of WSL was lower in ethanol/H_2_O than that in 1, 4‐dioxane/H_2_O (entries 4,5). It is possibly attributed to the formation of colloidal suspension of lignin during washes, which would not clear even after prolonging centrifugation. The formation of this colloidal lignin has also been reported (Pepper & Siddiqueullah, 1961). Yield and purity of WSL at 15.2 and 91.8 wt% were observed in the presence of 1, 4‐dioxane/H_2_O (entry 5). Compared to H_2_SO_4_, the lignin content was lower while an obvious difference in the yield was observed in the presence of HCl (entries 5,6). We speculated that Cl^−^ possibly interacted with hydroxyl of cellulose and/or lignin, thus promoting the dissolution of lignin and giving negative effect on precipitation. The performance of Amberlyst cation resins was also investigated. As shown in Table 2, both the yield and WSL purity were close to those obtained in the presence of strong inorganic acids (entries 5,7–9). These results showed that Amberlyst resins were effectively for the organosolv process of wheat straw. However, it was found that tiny amount of Amberlyst‐35 and most Amberlyst‐15 broke after organosolv process. It could be ascribed to their poor heat resistance at high temperature of 180°C, leading to difficult separation due to mix with cellulose. Amberlyst‐45 could be recovered and reused without any pretreatment. The results in Table 1 showed that Amberlyst‐45 displayed excellent stability, almost no change in the yield and content was observed (entries 7, 10).

**Table 1:** Isolation of lignin from wheat straw via organosolv process

Entry	Solvent	Catalyst	WSL	
			Yield (%)	Lignin content (wt%)
1^a^	Methanol	H_2_SO_4_	9.9	61.4
2^a^	Acetonitrile	H_2_SO_4_	11.7	62.5
3^a^	Acetone	H_2_SO_4_	14.1	64.1
4^a^	Ethanol	H_2_SO_4_	13.6	73.6
5^a^	1, 4‐Dioxane	H_2_SO_4_	15.2	91.8
6^a^	1, 4‐Dioxane	HCl	12.3	90.6
7	1, 4‐Dioxane	Amberlyst‐45	15.0	91.4
8	1, 4‐Dioxane	Amberlyst‐35	14.7	91.6
9	1, 4‐Dioxane	Amberlyst‐15	14.4	92.3
10^b^	1, 4‐Dioxane	Amberlyst‐45	14.5	91.7

Note

Reaction conditions: 10 g EWS, 100 ml 1, 4‐dioxane/H_2_O mixture (2:1, v/v), 5 g Amberlyst‐45, 180°C for 2.5 hr.

^a^ 1.5 wt% catalyst was employed.

^b^ The reusability Amberlyst‐45.

**Table 2:** Oxidative conversion of WSL

Entry	Oxidant	Yield (%)							Total yield (%)^f^
		Syringaldehyde	Vanillin	*p*‐Hydroxybenzaldehyde	Acetosyringone	Acetovanillone	Syringic acid	Vanillic acid	
1	Atmospheric pressure air	0.7	0.6	–	2.6	–	1.2	–	5.1
2^a^, ^b^	CuSO_4_.5H_2_O	0.6	0.5	–	1.4	–	0.6	–	3.1
3^b^	Nitrobenzene	3.0	2.5	0.3	0.4	–	1.6	0.3	8.1
4^a^	CuSO_4_.5H_2_O/air	1.6	1.4	0.1	3.4	0.2	0.8	0.1	7.6
5	Nitrobenzene/air	3.8	3.3	–	0.6	0.2	0.9	0.8	9.6
6^c^	Nitrobenzene/air	0.9	0.8	–	0.3	0.3	0.7	0.3	3.3
7^d^	Nitrobenzene/air	3.8	3.5	–	0.7	0.3	0.8	0.8	9.9
8^e^	Nitrobenzene/air	0.3	4.2	–	–	0.1	–	0.3	4.9

Note

Reaction conditions: 1 g WSL, 50 ml 2 M NaOH aqueous solution, 4 ml nitrobenzene, 170°C for 2.5 hr.

^a^ 5 mmol CuSO_4_.5H_2_O was employed.

^b^ Air in the autoclave was replaced by 1 MPa nitrogen before oxidation.

^c^ EWS as substrate, yield = product mass/(EWS mass × 0.201).

^d^ Purified lignin as substrate. The purified lignin was obtained according to the reported procedure (Jin et al., 2010).

^e^ Dealkaline lignin as substrate.

^f^ The sum of the yields of all detected monophenolic compounds (similarly hereinafter).

#### 3.2.2 Composition of wheat straw and lignin

**Figure 4:**
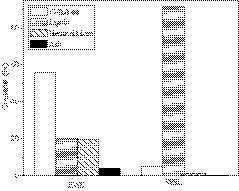
Composition of wheat straw and its derived sample

The compositions of EWS and WSL are presented in Figure 4. The contents of the three main components such as cellulose, lignin, and hemicellulose of EWS were 56.0, 20.1, and 19.5 wt%, respectively. In addition, ash content at 4 wt% was also determined. WSL with high purity of lignin at 91.4 wt% was obtained after organosolv process. Almost no ash was detected in WSL, and the contents of cellulose and hemicellulose of WSL were only 4.7 and 1.3 wt%, respectively. It is expected to be beneficial for further oxidative conversion. These results revealed that Amberlyst‐45 is an effective heterogeneous catalyst for the separation of lignin from wheat straw, giving lignin with high quality. The elemental analysis (EA) results and molecular weight averages of WSL were supplied by Tables S1 and S2.

### 3.3 Oxidative conversion of lignin isolated from wheat straw

#### 3.3.1 Investigation on oxidant

Atmospheric pressure air, oxygen, cupric(II), and nitrobenzene were usually employed for the oxidative conversion of lignin (Sun et al., 1995). The conversion of lignin derived from wheat straw was first investigated using atmospheric pressure air as oxidant due to its availability (entry 1). The results in Table 2 showed that the total yield of monophenolic compounds at 5.1% was achieved. The yield of aromatic aldehydes was relatively close, 0.7 and 0.6% for syringaldehyde and vanillin, respectively. Acetosyringone was the predominant product, giving yield at 2.6% but almost no acetovanillone was detected. Similarly, yield of syringic acid at 1.2% was acquired while almost no detection of vanillic acid. Obviously, the amount of the products derived from S units was larger than those from G and H units. Rahimi^9^ reported that products derived from lignin are very similar to monomer composition. So the amount of the construction units in WSL is possible as the following order: S units > G units > H units. It agreed with the ^1^H NMR spectrum shown in Figure 2, which suggests that WSL is mainly composed of S and G units. Contrarily, almost no products derived from H units could also be ascribed to its low content in WSL. The oxidative conversion of WSL was also investigated in the presence of CuSO_4_.5H_2_O (entry 2). The product composition was quite similar to that obtained in the presence of air. Acetosyringone still was the predominant product. The total yield of monophenolic compounds and yield of acetosyringone were 3.1 and 1.4%, respectively. Although both of syringaldehyde and acetosyringone were derived from S units, there was an obvious difference in the yields of them. It might be ascribed to the competitive cleavage of C_α_–C_β_ and β−O−4 linkages in lignin polymer (Evtuguin, Daniel, Silvestre, Amado, & Neto, 2000). It is believed that the former produces aromatic aldehydes, while the latter produces ketones with acetyl structure (Li et al., 2015).

The formation of monophenolic compounds from WSL via nitrobenzene oxidation procedure under alkaline condition has been widely reported (Ramezani & Sain, 2018;Schultz & &Templeton, 1986;Sun et al., 1995). Compared to atmospheric pressure air and CuSO_4_.5H_2_O, the total yield of monophenolic compounds increased significantly, and yield at 8.2% was observed (entry 3). A small amount of *p*‐hydroxybenzaldehyde also yielded, meaning the oxidation of WSL took place more completely. These results indicated that nitrobenzene possessed stronger oxidative property for lignin conversion. Although the total yield increased, the yield of acetosyringone dropped more sharply compared to those obtained in the presence of atmospheric pressure air and CuSO_4_.5H_2_O, only 0.4% was observed. Furthermore, close yields at 3.0 and 2.5% for syringaldehyde and vanillin were achieved, respectively. The formation of syringic and vanillic acids was also promoted, giving yields at 1.6 and 0.3%, respectively. These results revealed that the formation of aromatic acid depended on the amount of aromatic aldehyde. The results in Table 2 displayed that derivatives from S units were the major products invariably. However, the amount of the as‐formed products changed by varying oxidant. This is inconsistent with the work reported by Besson et al. (2014), in which vanillin is usually the predominant product. It possibly could be attributed to lignin source. The obvious differences in the product distribution suggested that the cleavage of β −O−4 linkages may be not the main route for the oxidative conversion of lignin using nitrobenzene as oxidant. It is different from that in the presence of air and CuSO_4_.5H_2_O. Table 2 revealed that the formation of aromatic compounds depended on oxidant significantly. So we speculate that the conversion of lignin undertakes different pathways in the presence of various oxidants.

It also can be seen from Table 2 that the formation of syringic acid cannot be neglected, especially in the presence of nitrobenzene. To improve the selectivity of aromatic aldehydes, the combined oxidants were further employed. Amazingly, the total yield was increased whether by combining CuSO_4_.5H_2_O or nitrobenzene with atmospheric pressure air (entries 4, 5). Higher yield was obtained in the presence of nitrobenzene/air than that of CuSO_4_.5H_2_O/air. Total yield at 7.6% was achieved in the presence of CuSO_4_.5H_2_O/air. The yield of acetosyringone reached 3.4%, which was higher than that obtained using CuSO_4_.5H_2_O or air as the single oxidant. The improvement in the production of acetosyringone further confirmed the cleavage of β − O−4 linkages in lignin polymer using CuSO_4_.5H_2_O and/or air as oxidant. The total yield was increased to 9.6% using nitrobenzene/air as composite oxidant. Close yields of syringaldehyde and vanillin at 3.8 and 3.3% were acquired. However, the yield of syringic acid at 0.9% dropped obviously compared to those obtained in the presence of single oxidant. Both the total yield and product composition were closer to those obtained using nitrobenzene as the single oxidant. So we speculated that air played the role of co‐oxidant. The drop in the yield of syringic acid possibly ascribed to the inhibition of deep oxidation of aromatic aldehydes, resulting from the competition oxidation of air and nitrobenzene.

The oxidation of EWS was also investigated in the presence of nitrobenzene and atmospheric pressure air (entry 6). Poor performance was observed, giving 3.3% total yield. It was much lower than that of WSL, confirming that there are interactions among the main constituents of lignocellulose, which reduces conversional property of lignin. Combining the results listed in Figure 4, it could be ascribed to the removal of residual cellulose and hemicellulose from WSL, resulting in the enhancement in the purity of lignin and drop in the interaction among components of lignocellulose. The oxidative conversion of purified lignin (95.6 wt% lignin purity) was also performed. The product distribution is similar to that of WSL, and the total yield increased from 9.6% to 9.9% slightly (entry 7). These results indicated that lignin obtained by organosolv fractionation is suitable to directly convert due to little difference in the yields of aromatic compounds derived from WSL and purified lignin.

Dealkaline lignin is the most common and available since extensive pulp and paper production via alkali papermaking worldwide. Therefore, the oxidative conversion of commercial dealkaline lignin was also investigated herein (entry 8). The total yield at 4.9% was obtained using nitrobenzene/air as combined oxidant. Vanillin was the predominant product, giving 4.2% yield. Only a small amount of syringaldehyde with 0.3% yield was detected. In addition, a small amount of vanillic acid while no syringic acid was detected, further indicating that the formation of aromatic acid was associated with the amount of aromatic aldehyde. The results in Table 2 indicated that aromatic aldehydes were the predominant products whether using dealkaline lignin or WSL as substrate in the presence of nitrobenzene/air (entries 5,8). However, obvious difference in product composition and species was observed. The products derived from G units played the main role for the oxidative conversion of dealkaline lignin. ^13^C NMR spectrum of dealkaline lignin confirms the presence of ferulic acid (see SI). It could be ascribed to the cracking of part linkages of G units in lignin polymer, which results in oligomers and thus favors for the subsequent transformation. Furthermore, almost no production of acetosyringone was detected using dealkaline lignin as substrate. It is possibly associated with the small quantity of β − O−4 linkages in dealkaline lignin, as NMR spectra shown in Figures S1 and S2.

#### 3.3.2 Screening of catalyst

It has been reported that acid, base, and salen complex are widely employed for the conversion of lignin (Li et al., 2015). Therefore, the conversion of WSL was investigated by varying catalyst, as shown in Table 2. H_3_PW_12_O_40_ revealed poor performance although acceptable performance of heteropolyacid for the conversion of Kraft lignin into vanillin and methyl vanillate was reported (Werhan et al., 2011). The total yield was almost negligible, only 0.6% was given (entry 1). NaAlO_2_ is water soluble and has strong basicity. Thus, it is widely used as alkaline catalyst in some reactions (Wan, Yu, Wang, & Luo, 2009). Herein, it was also employed in the oxidative conversion of WSL. The results in Table 3 showed that the total yield of monophenolic compounds at 2.4% was obtained (entry 2). Monophenolic compounds’ yield at 5.9% was observed when strong base of CsOH was employed as catalyst (entry 3). It was much lower than that of 9.6% obtained in the presence of NaOH (entry 4). These results were inconsistent to the alkalinity of the alkaline catalysts. Thus, it is speculated that the formation of monophenolic compounds is related to the alkalinity in terms of variation in the yield. Nevertheless, moderate alkalinity is possible more helpful to the conversion of WSL to monophenolic compounds. Total yields at 7.1 and 3.2% were acquired in the presence of 2.5 and 1 M NaOH aqueous solution further supported this point (entries 5, 6).

**Table 3:** Conversion of WSL with various catalysts^a^

Entry	Catalyst	Amount of catalyst	Yield (%)							Total yield (%)
			Syringaldehyde	Vanillin	*p*‐Hydroxybenzaldehyde	Acetosyringone	Acetovanillone	Syringic acid	Vanillic acid	
1	H_3_PW_12_O_40_	0.5 g	0.2	0.1	–	0.1	–	0.2	–	0.6
2	NaAlO_2_	2 M	0.9	0.8	0.2	0.3	–	0.2	–	2.4
3	CsOH	2 M	2.6	2.2	–	0.4	0.2	0.3	0.2	5.9
4	NaOH	2 M	3.8	3.3	–	0.6	0.2	0.9	0.8	9.6
5	NaOH	2.5 M	2.5	1.9	0.5	0.5	0.2	0.7	0.8	7.1
6	NaOH	1 M	1.1	0.8	–	0.2	–	0.9	0.2	3.2
7	H_3_PW_12_O_40_/NaOH	0.5 g + 2 M	1.2	0.8	0.5	0.5	–	1.6	0.5	5.1
8	NaOH/NaAlO_2_	1.5M + 0.5 M	2.2	1.9	0.7	0.4	–	2.5	0.9	8.6
9^b^	NaOH/NaAlO_2_	1.5 M + 0.5 M	4.4	3.9	0.3	0.9	0.4	1.3	1.1	12.3
10^c^	NaOH/NaAlO_2_	1.5 M + 0.5 M	3.3	2.8	0.2	0.3	0.2	0.8	0.6	8.2
11^b^	NaOH/NaAlO_2_	1.25 M + 0.75 M	5.3	5.0	0.4	1.2	0.4	1.3	1.5	15.1
12^b^	NaOH/NaAlO_2_	1.0 M + 1.0 M	6.0	5.6	0.5	1.1	0.6	2.0	1.5	17.3
13^b^	NaOH/NaAlO_2_	0.75 M + 1.25 M	6.3	5.7	0.7	1.3	0.6	1.9	1.6	18.1
14^b^	NaOH/NaAlO_2_	0.5 M + 1.5 M	4.5	4.1	0.6	1.1	0.5	1.3	0.9	13.2
15^d^	NaOH/NaAlO_2_	1.0 M + 1.0 M	0.6	0.4	0.6	2.4	0.2	0.6	0.1	4.9

Note

Reaction conditions: 1 g WSL, 50 ml aqueous solution, 4 ml nitrobenzene, 170°C for 2.5 hr.

^a^ The air in the autoclave was not replaced before reaction.

^b^ NaAlO_2_ calcined at 150°C.

^c^ NaAlO_2_ calcined at 300°C.

^d^ No nitrobenzene was added.

The oxidative conversion of WSL was further investigated by combing H_3_PW_12_O_40_ and NaOH (entry 7), and the total yield of monophenolic compounds at 5.1% was obtained. It decreased obviously compared to that given by using NaOH as single catalyst. However, the deep oxidation of WSL was enhanced in the presence of acid. Syringic acid became the predominant product, giving 1.6% yield. The combination of NaOH and NaAlO_2_ was also investigated. The total yield was between those given by employing NaOH and NaAlO_2_ as single catalyst. Only a little decrease in the total yield was observed compared to that of NaOH, giving total yield at 8.6% (entry 8). Similar to H_3_PW_12_O_40_/NaOH, syringic acid was the main product. The results in Table 3 revealed that the conversion of WSL was improved apparently once NaAlO_2_ was calcined before reaction (entry 9). Amazingly, the total yield increased from 9.6% to 12.3% via calcination of NaAlO_2_ at 150°C. Furthermore, syringaldehyde and vanillin were the predominant products, giving yields at 4.4 and 3.9%, respectively. However, calcination of NaAlO_2_ at higher temperature resulted in the reduction in the catalytic activity. The total yield decreased to 8.2% (entry 10) possible due to phase transition of NaAlO_2_ (Mutreja, Singh, & Ali, 2012).

The effect of component amounts in composite catalysts was also investigated. The results in Table 3 showed that the distribution of the product was almost independent on the ratio of catalyst (entries 9, 12–14). Syringaldehyde and vanillin still were the main products. The total yield increased gradually as the decrease in the amount of NaOH. When both the amounts of NaOH and NaAlO_2_ were 1 M, the total yield reached 17.3%. Then, the total yield almost kept constant with decrease in the amount of NaOH. Further drop in the amount of NaOH led to decrease in the total yield once NaOH concentration below 0.75 M. These results further confirmed that the conversion of WSL required moderate alkalinity. In addition, the total yield was only 4.9% in the absence of nitrobenzene (entry 15), and acetosyringone was the main product. The product distribution is similar to the results shown in Table 2 using atmospheric pressure air as oxidant singly (Table 2, entry 1), further confirming that the product composition depended oxidant extremely. The effect of oxidant on the conversion of WSL became more significant in the presence of NaAlO_2_. The total yield increased from 6.9% to 17.3% (entries 12, 15) using nitrobenzene/air as oxidant. It is more obvious than that changed from 5.1% to 9.6% using NaOH only (Table 2, entries 1, 5).

The results in Table 3 revealed that the highest total yield of aromatic compounds and aromatic aldehydes were acquired in the presence of nitrobenzene/air. Therefore, the product was further analyzed by GC‐MS and LC‐HRMS (see SI, Figures S3–S15). The formation of *p*‐hydroxybenzaldehyde, vanillin, syringaldehyde, acetovanillone, acetosyringone, vanillic acid, and syringic acid was further confirmed. In addition, a large number of oligomers with molecular weight > 225 were also detected by LC‐HRMS, which were possibly ascribed to the repolymerization of monophenolic compounds from oxidation and/or conversion of lignin. Although the yield of lignin at 18.1% was obtained, it is still far below theoretical conversion. This possible could be explained by the formation of oligomers.

#### 3.3.3 Comparison of the oxidative conversion of lignin

The oxidative conversion of lignin has been widely investigated. The woks related to the conversion of lignin were compared, as shown in Table 4. In this work, the total yield of monophenolic compounds at 18.1% was obtained (entry 1) suing NaOH/NaAlO_2_ as composite catalyst. It was much higher than those obtained using H_2_O_2_, O_2_, or benzoquinone compound (DDQ) as oxidant (entries 2–8). Partenheimer (Partenheimer, 2009) reported 10.9% total yield of aromatic benzaldehyde and carboxylic acid (entry 9). However, both the catalytic system and product composition were too complicated. The results in Table 4 also indicated that lignin isolated from wheat straw revealed much higher activity than that obtained using wheat straw as substrate under identical conditions (Sun et al., 1995), further confirming it is essential to isolate lignin from straw to obtain effective transformation.

**Table 4:** Comparison in oxidative conversion of lignin in various works

Entry	Substrate	Catalyst/Oxidant	Predominant product	Total yield (%)	References
1	Wheat straw lignin	NaOH/NaAlO_2_/Nitrobenzene‐air	Vanillin, syringaldehyde	18.1	This work
2	Kraft lignin	Nb_2_O_5_/H_2_O_2_	Vanillin, acetovanillone	0.4	Das et al. (2017)
3	Sugar beet pulp	CuSO_4_/O_2_	Vanillin, vanillic acid	1.4	Aarabi et al. (2017)
4	Kraft lignin	H_3_PMo_12_O_40_/O_2_	Vanillin, methyl vanillate	5.2	Voitl and Von Rohr (2008)
5	Wheat straw	NaOH/Nitrobenzene	*p*‐Hydroxybenzaldehyde, vanillin, syringaldehyde	5.7	Sun et al. (1995)
6	Lignin	NaOH/O_2_	Vanillin	7.6	Araújo, Grande, and Rodrigues (2010)
7	Kraft lignin	CoCl_2_/O_2_	Vanillin, methyl vanillate	6.3	Werhan, Mir, Voitl, and Von Rohr (2011)
8	Kraft lignin	H_3_PMo_12_O_40_/O_2_	Vanillin, methyl vanillate	8.8	Voitl, Nagel, and Von Rohr (2010)
9	Lignin organosolv	Co‐Mn‐Zr‐Br/O_2_	Aromatic benzaldehyde, carboxylic acid	10.9	Partenheimer (2009)

## 4 CONCLUSIONS

A green methodology for extracting lignin from wheat straw and an effective catalytic system for the oxidative conversion of WSL were developed. Amberlyst‐45 and composite catalyst composed of NaOH/NaAlO_2_ were effective for WSL extraction and oxidative conversion, respectively. WSL with purity at 91.4 wt%, mainly composed of S and G units, was obtained via organosolv process using Amberlyst‐45 as green and recyclable catalyst. The conversion of lignin was significantly enhanced via organosolv fractionation in terms of total yield of monophenolic compounds. The total yield of monophenolic compounds was enhanced from 3.3% to 9.6% in NaOH solution using nitrobenzene/ atmospheric pressure air as composite oxidant. Both the formation of monophenolic compounds and aromatic aldehydes were also enhanced by combining oxidants and catalyst. The total yield of monophenolic compounds reached up 18.1%, and yields at 6.3 and 5.7% of syringaldehyde and vanillin were achieved in the presence of nitrobenzene/atmospheric pressure air and NaAlO_2_/NaOH.

## CONFLICT OF INTEREST

All authors declare no conflicts of interest.

## ETHICAL STATEMENT

This study does not involve any human or animal testing.

## Supporting information

Supinfo
