# Supplementary material for: Oxidative conversion of lignin isolated from wheat straw into aromatic compound catalyzed by NaOH/NaAlO2
Source: Food Sci Nutr. 2020 May 22;8(7):3504–14. doi: 10.1002/fsn3.1633 (PMC7382187; doi:10.1002/fsn3.1633)
Supplement: Supplementary file 1 — Supinfo [file FSN3-8-3504-s001.docx]

**Oxidative conversion of lignin isolated from wheat straw via organosolv process into aromatic compound**

**Kehui Luo^1^** | **Sijiu Zhao^1^** | **Guozhi Fan^1^** | **Qunpeng Cheng^1^** | **Bo Chai^1^** | **Guangsen Song^1^**

School of Chemical and Environmental Engineering, Wuhan Polytechnic University, Wuhan 430023, Hubei, China

**1  |  METHODS**

**1.1 | Materials**

Elemental analysis (EA) of C, H and N were performed on a Vario EL III elemental analyzer. Nuclear magnetic resonance (NMR) including ^1^H NMR and ^13^C NMR spectra were acquired on a Unity-Inova 600 at 50^o^C using DMSO-d6 as solvent and tetramethylsilane as internal standard. Thermogravimetry analysis (TGA) was performed using a Perkin-Elmer TG-DSC 7 spectrometer under nitrogen at a heating rate of 10◦C/min in the range from room temperature to 800◦C. Approximately 10 mg of samples were used in each analysis and the gas ﬂow rate was kept at 90 mL/min.

**1.2 | Qualitatively analysis**

The product derived from wheat lignin was qualitatively analysis by Liquid chromatography-high resolution mass spectrometry (LC-HRMS) and gas chromatography mass spectrometry (GC-MS). LC-HRMS was performed on a UFLC-20A [high-performance liquid chromatography](http://www.baidu.com/link?url=X08yiIPk00f22N8HkIJg8Dki1z-jd0omqCbB_Uxa0R4_tuBnRGAuCxsdtbsGufMwkzgSfniGYhPNF4qDMBlesisQRwrmOaq7dvoNiLGXSue) (Shimadzu, Kyoto, Japan) and AB Sciex TripleTOF 5600 mass spectrometer equipped with an inverse Agilent XDB-C18 column (2.1 × 100 mm; 3.5 μm). The detection was performed in the scan range from m/z of 30 to 500. GC-MS was performed on a Varian 450 GC-320 MS equipped with VF-5 MS capillary column (30 m × 0.32 mm × 0.25 μm) and EI source. The initial temperature was 50^o^C and retained 2 min, then ramped to 250^o^C at 5^o^C/min and retained for 2 min. The detection was performed in the scan mode from m/z 20 to 500, and 1.0 mL/min helium was used as the carrier gas. The ionization voltage and source temperature was 70 eV and 280^o^C, respectively.

**2 | CHRACTERIZATION AND ANALYSIS**

**2.1 | EA**

**TABLE s1** EA results of wheat straw lignin (WSL)

| Sample | Content (wt%) | | |
| --- | --- | --- | --- |
|  | C | H | O |
| WSL | 62.03 | 5.315 | 32.654 |
| Dealkalized lignin | 49.91 | 4.730 | 45.359 |

**2.2 | Molecular weight of lignin isolated from wheat straw**

**TABLE s2** Molecular weight averages of lignin isolated from wheat straw

| Sample | M_w_ (g/mol) | M_n_ (g/mol) | D |
| --- | --- | --- | --- |
| WSL | 2189 | 1115 | 1.96 |

**2.3 | ^1^H NMR**

**FUGURE S1** ^1^H NMR spectrum of dealkalinized lignin

**2.4 | ^13^C NMR**

**FIGURE S2**  ^13^C NMR spectrum of dealkalized lignin

**2.5 | GC-MS**

**FIGURE S3** GC-MS of vanillin

**FIGURE S4** GC-MS of syringaldehyde

**FIGURE S5** GC-MS of acetosyringone

**2.6 | LC-HRMS**

**FIGURE S6**  LC-HRMS of *p*-hydroxybenzaldehyde


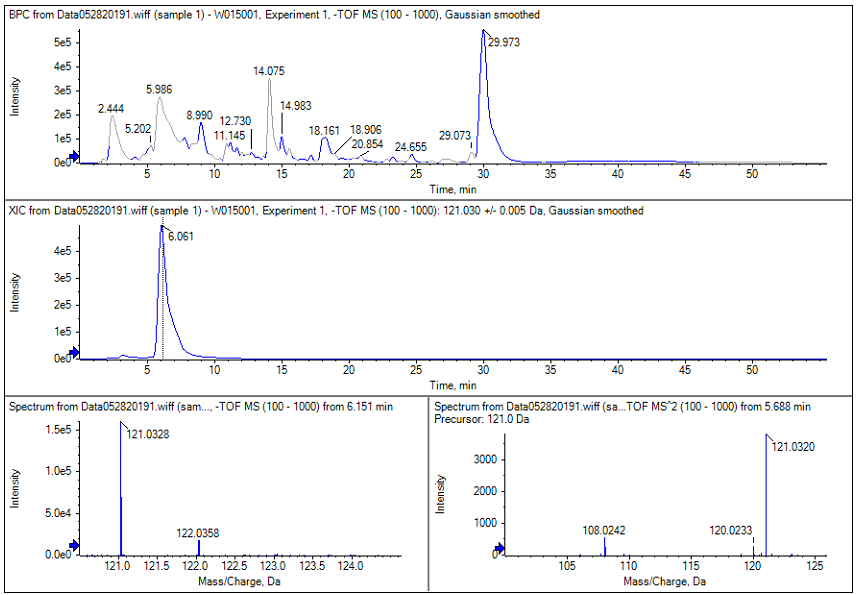


**FIGURE S7**  LC-HRMS of vanillin


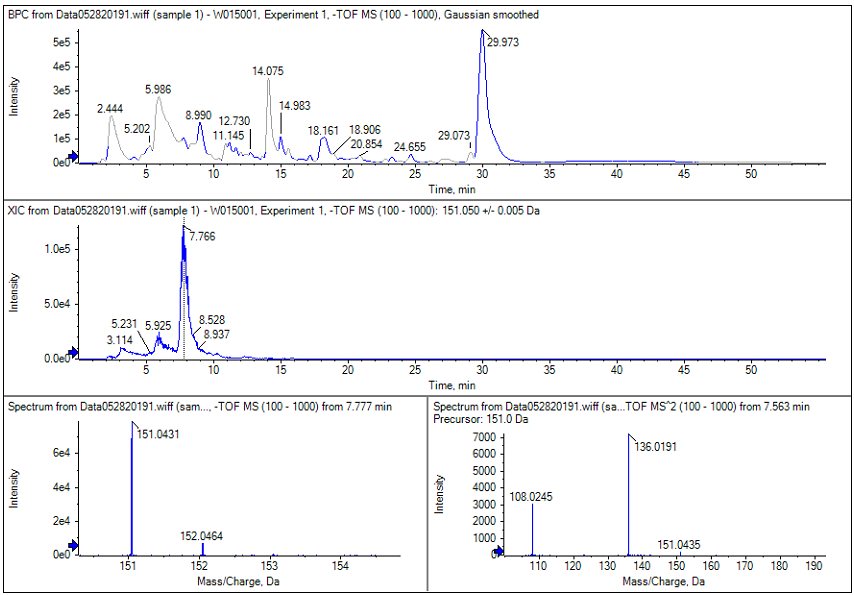


**FIGURE S8**  LC-HRMS of syringaldehyde


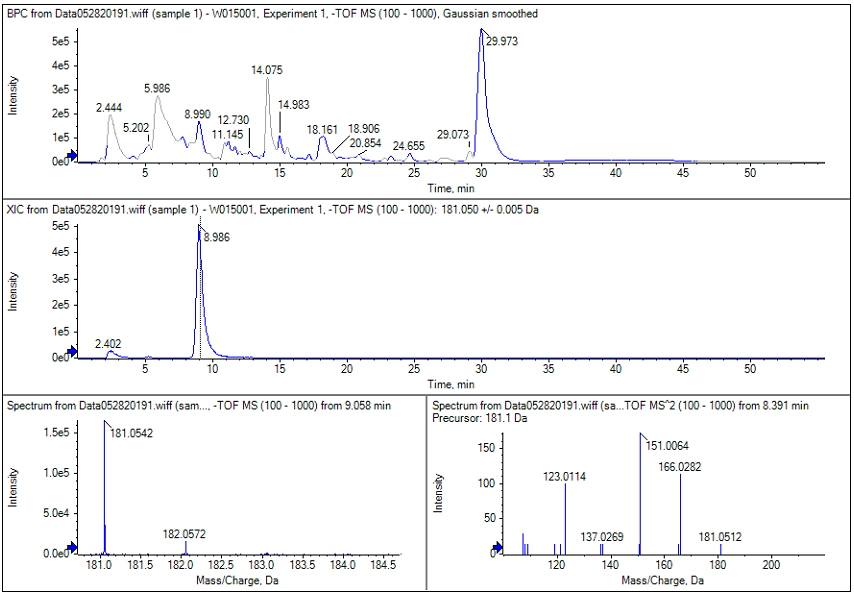


**FIGURE S9**  LC-HRMS of acetovanillone


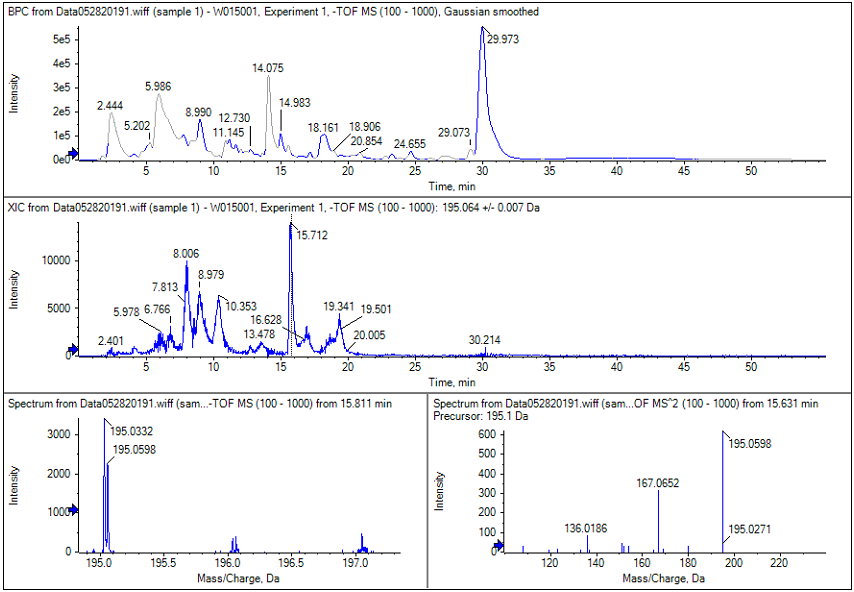


**FIGURE S10**  LC-HRMS of acetosyringone


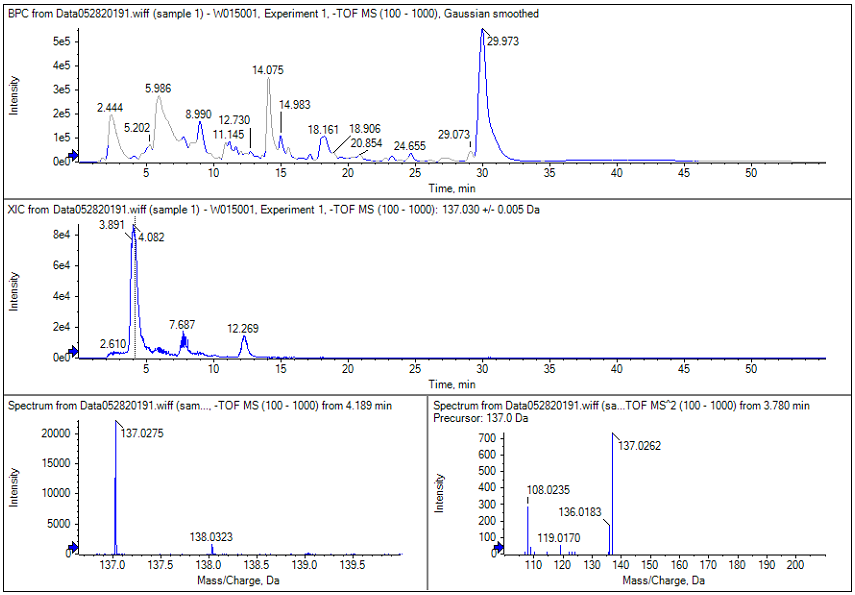


**FIGURE S11**  LC-HRMS of *p*-hydroxybenzoic acid


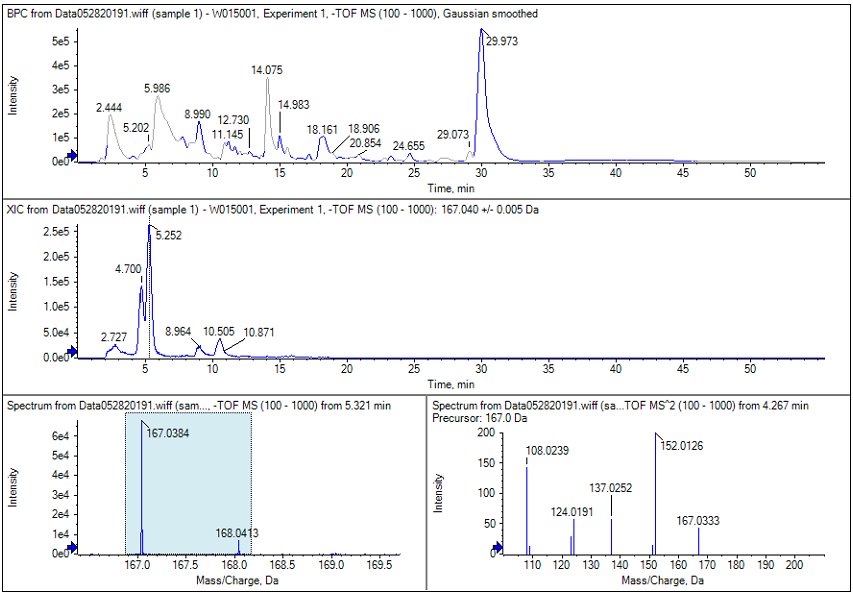


**FIGURE S12**  LC-HRMS of vanillic acid


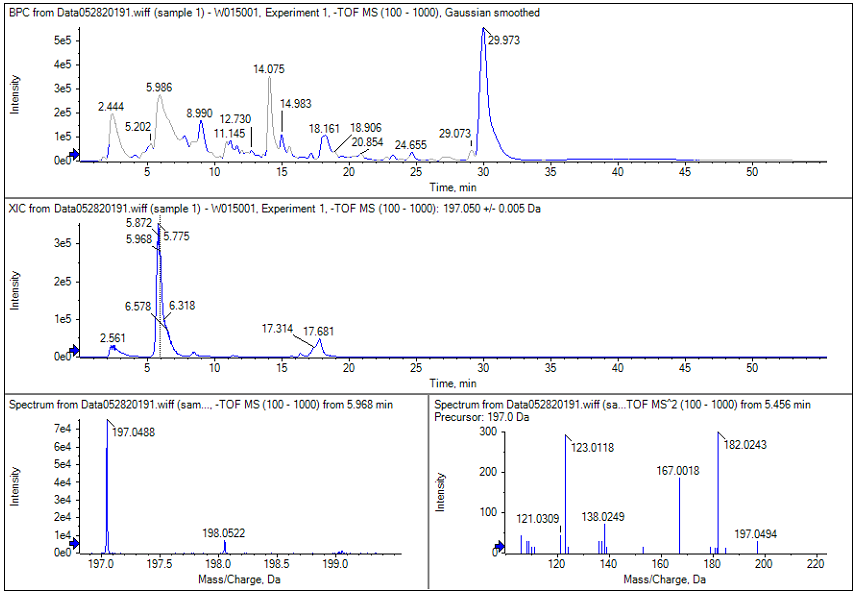


**FIGURE S13**  LC-HRMS of syringic acid


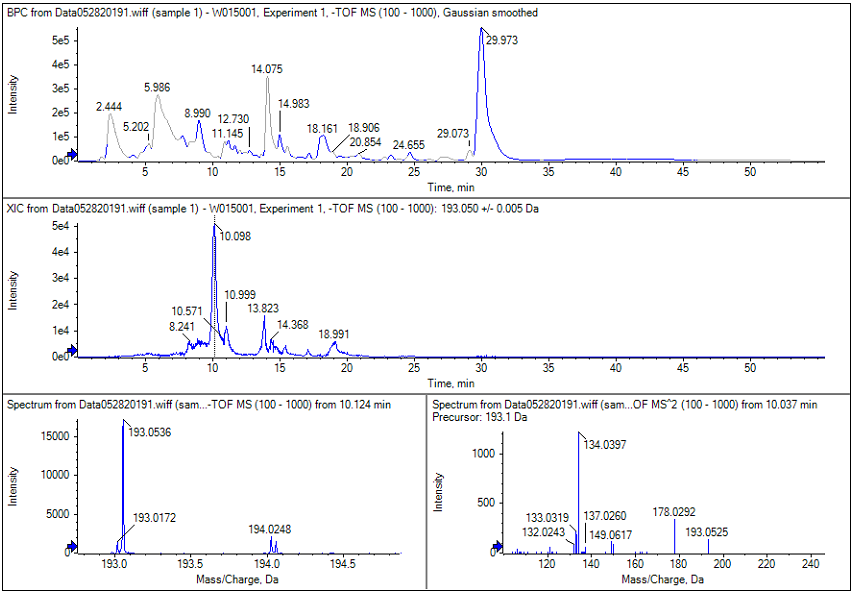


**FIGURE S14**  LC-HRMS of ferulic acid


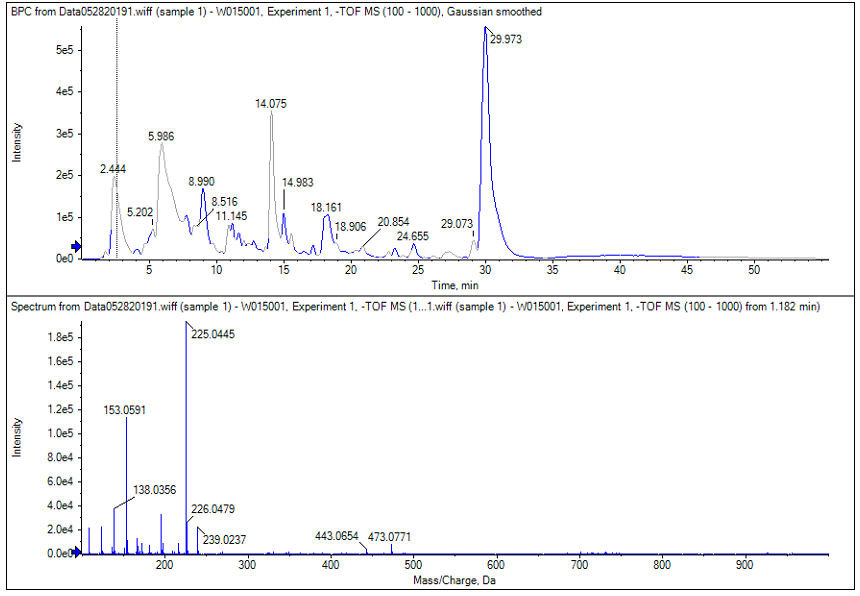

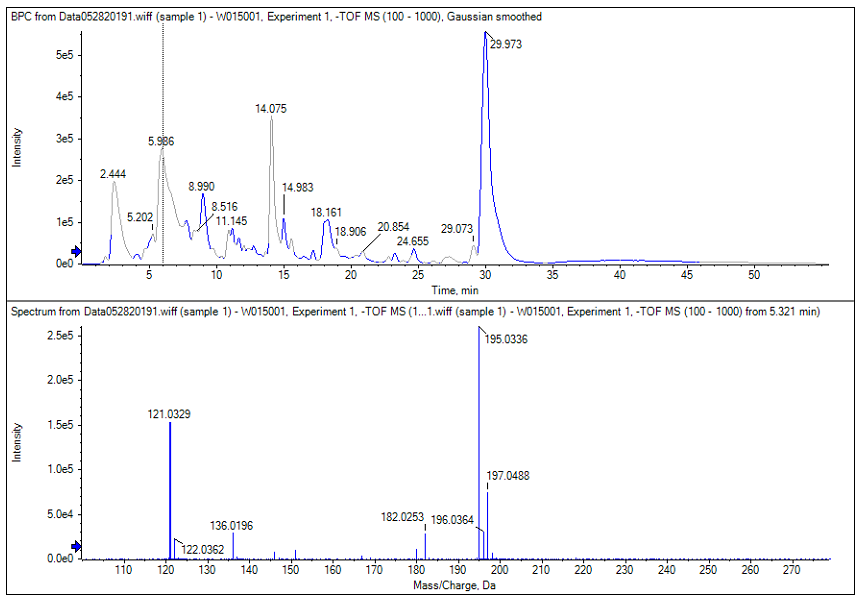

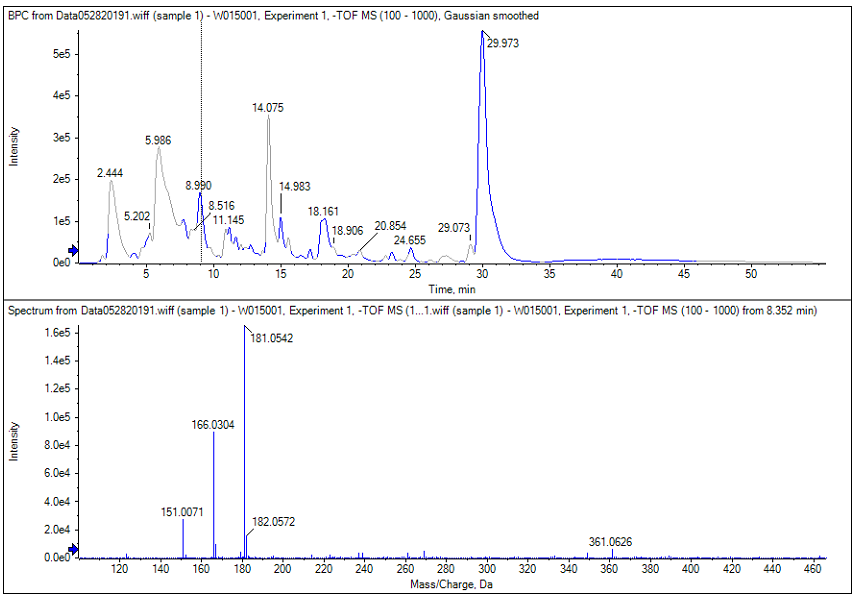

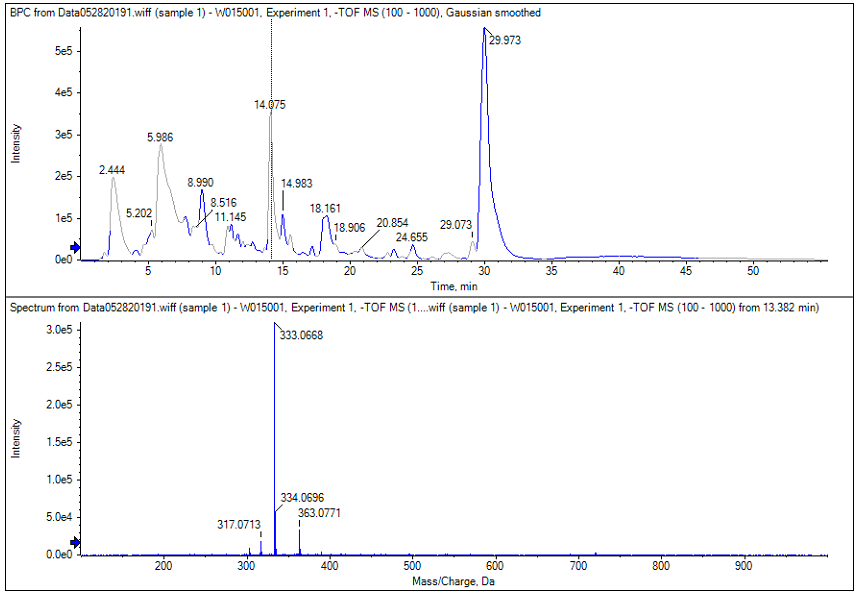


**FIGURE S15**  LC-HRMS of oligomers
